# A case report of fungal infection associated acute fibrinous and organizing pneumonitis

**DOI:** 10.1186/s12890-020-1145-7

**Published:** 2020-04-20

**Authors:** Jiangnan Zhao, Yi Shi, Dongmei Yuan, Qunli Shi, Weiping Wang, Xin Su

**Affiliations:** 1 Department of Respiratory and Critical Care Medicine, Jinling Hospital, Medical School of Nanjing University, Nanjing, 210002 China; 2 Department of Pathology, Jinling Hospital, Medical School of Nanjing University, Nanjing, 210002 China; 3 Department of Clinical Laboratory, Jinling Hospital, Medical School of Nanjing University, Nanjing, 210002 China

**Keywords:** Acute fibrinous and organizing pneumonitis, *Penicillium citrinum*, Infection, Case report

## Abstract

**Background:**

Acute fibrinous and organizing pneumonitis (AFOP) is an uncommon variant of acute lung injury, characterized by intra-alveolar fibrin and organizing pneumonia. Proposed etiologies include connective tissue diseases, infections, occupational exposure, drug reactions, and autoimmune disease. Here we present a rare case of fungal infection associated AFOP in patient with diabetes mellitus (DM) and review the relevant literature.

**Case presentation:**

A 67-year-old man complained of cough, fever, dyspnea and hemoptysis. Patient experienced a rapidly progressive course exhibit diffuse predominant consolidation, ground glass opacities, and multifocal parenchymal abnormalities on chest computed tomography (CT). Antibacterial, antifungal, and antiviral treatments were ineffective. A CT-guided percutaneous lung biopsy was performed. Histologically, the predominant findings were as follows: alveolar spaces filled with fibrin and organizing loose connective tissues involving 70% of the observed region, pulmonary interstitial fibrosis, and small abscesses and epithelioid cell granuloma in the focal area. Result of periodic acid-silver methenamine stain was positive. The fungal pathogen from the sputum culture was identified as *P. citrinum* repeatedly over 3 times. Patient was diagnosed with DM during hospitalization. Corticosteroids combined with an antifungal therapy were effective. Follow-up for 4 months showed complete radiological resolution.

**Conclusions:**

As this common “contaminant” can behave as a pathogen in the immunocompromised host, both clinicians and microbiologists should consider the presence of a serious and potentially fatal fungal infection on isolation of *P. citrinum*. Based on this case, it could be speculated that AFOP may be associated with fungal infection including *P. citrinum*.

## Background

Acute fibrinous and organizing pneumonitis (AFOP) is a rare form of idiopathic interstitial pneumonia (IIP), which has currently been increasingly recognized and added to the American Thoracic Society/European Respiratory Society (ATS/ERS) international multidisciplinary classification of IIP [1]. AFOP is a rare histopathological diagnosis, which is characterized by organized intra-alveolar fibrin. AFOP can be idiopathic or associated with known causes, such as connective tissue disorders, drugs, occupational exposure, immune system disorders, and infections [2–5], requiring prompt clinical evaluation. Clinical characteristics associated with this disease are non-specific and vary widely. To the best of our knowledge, there have been no reports on AFOP associated with fungal infections. We present a case of AFOP combined with fungal infection, which presented with a rapidly progressive clinical course and radiological findings, and responded well to corticosteroids combined with an antifungal treatment.

## Case presentation

A 67-year-old man, former smoker (20 pack-year), complained of cough with white mucous sputum for over 2 weeks and developed fever for 3 days. The medical history included hypertension, which was well-controlled with nifedipine.

**Fig. 1:**
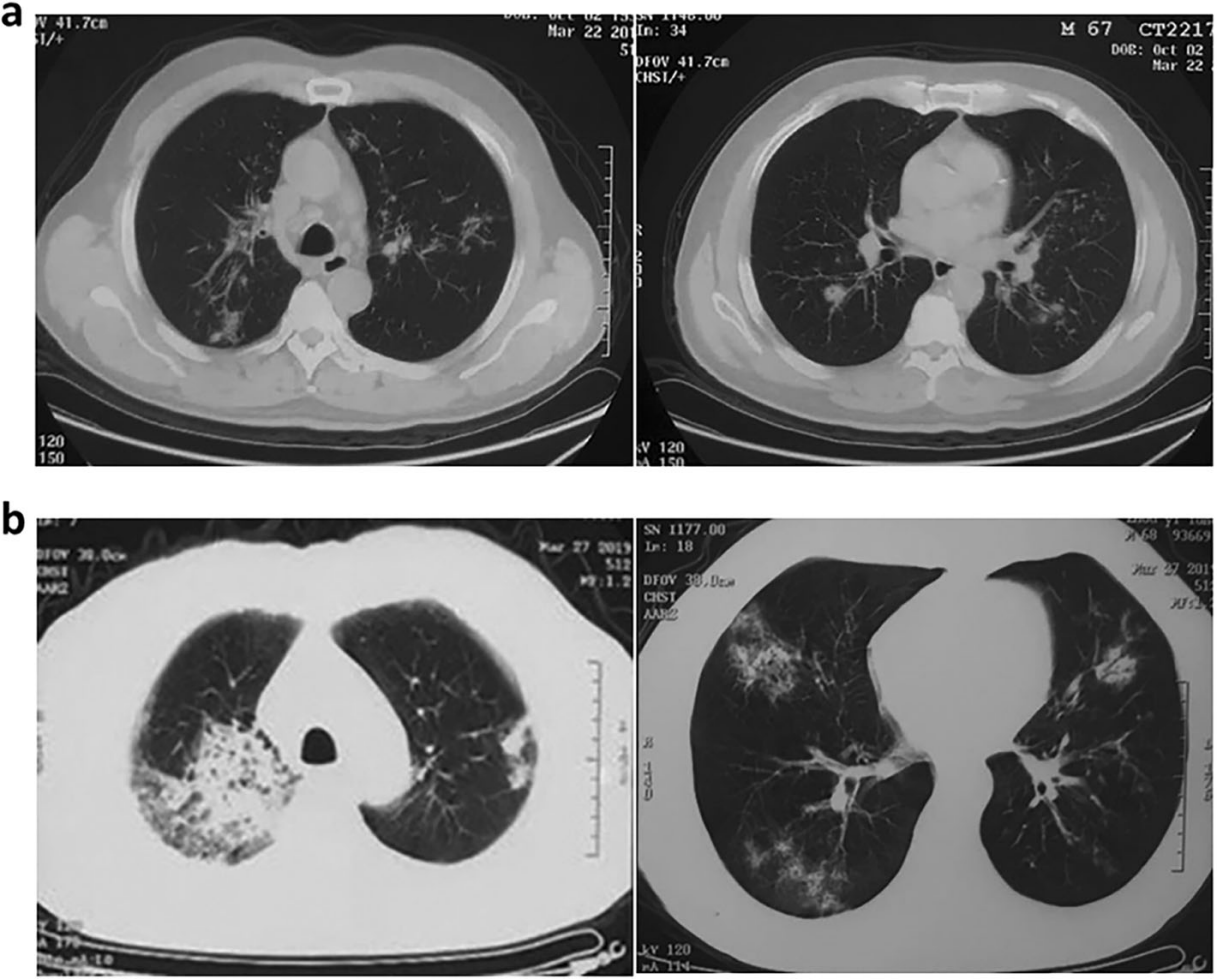
**a** CT on March 22 showing bilateral diffuse ground-glass opacities and multi-focal, patchy, ill-defined nodular opacities in the lungs. **b** Newly developed multi-focal dense consolidations are observed

Blood investigations at a local hospital revealed a white blood cell (WBC) count of 19.17 × 10^9^/L, and markedly elevated C-reactive protein (CRP) level (242.26 mg/L). Initial chest computed tomography (CT) on March 22 showed bilateral scattered consolidation areas **(**Fig. 1a**)**. Empirical therapy was provided for community-acquired pneumonia (CAP) with piperacillin-sulbactam, moxifloxacin and ceftriaxone. However, he showed no signs of improvement. Laboratory investigations revealed a positive serum galactomannan (GM) antigen test (1.76), and *Aspergillus* was isolated from the sputum culture. The patient was treated with fluconazole, but the treatment was ineffective. Chest radiography on March 27 revealed obviously increased bilateral parenchymal opacities **(**Fig. 1b**)**. As the patient’s condition further deteriorated, he was transferred to the Department of Respiratory and Critical Medicine at Jinling Hospital.

On admission, his vital signs were as follows: body temperature, 38.6 °C; pulse rate, 84 beats/min; respiratory rate, 18 breaths/min; and blood pressure, 129/74 mmHg and; oxygen saturation on room air, 95%. Chest auscultation revealed increased breath sounds with fine crackles and wheezing in the upper right lung zones, with no other remarkable findings. The abnormal laboratory test results were as follows: WBC count, 14.25 × 10^9^/L; neutrophils%, 81.8; CRP, 69.6 mg/L; albumin, 25.0 g/L; alanine aminotransferase, 109 U/L; procalcitonin, 0.105 μg/L; and interleukin-6, 224.60 ng/L. The autoimmune antibody profile, CD4 lymphocyte count, IgM, IgG, IgE and tumor biomarkers were within the normal limits. Other laboratory investigations, including rapid antigen tests for influenza A and B, the Mantoux test, and the T-spot test, were all negative. However, he had poorly controlled blood sugar during hospitalization. He received a diagnosis of diabetes mellitus (DM) type 2 from endocrinologist.

Based on the sputum culture, blood GM test, and CT at the local hospital, we initially diagnosed the patient with “probable” invasive pulmonary aspergillosis (IPA) and treated him with voriconazole. However, the patient’s clinical status worsened, with persistent fever. The serum GM test result at our hospital was negative. Fiberoptic bronchoscopy with bronchoalveolar lavage (BAL) was performed the following day. On admission day 4, the patient developed exertional dyspnea and hemoptysis. We suspected drug-resistant pneumonia and treated the patient empirically with anti-bacterial (biapenem, linezolid), anti-fungal (caspofungin), and anti-viral (oseltamivir, acyclovir) drugs in succession.

**Fig. 2:**
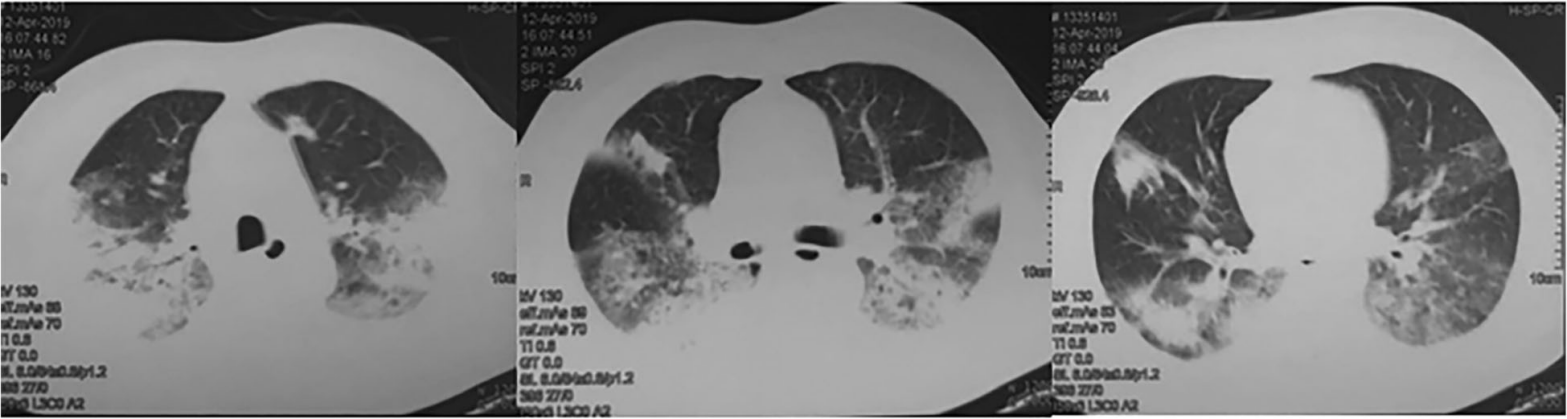
Bilateral diffuse patchy opacities, multi-focal dense consolidations and bronchial shadows in some lesions are observed

**Fig. 3:**
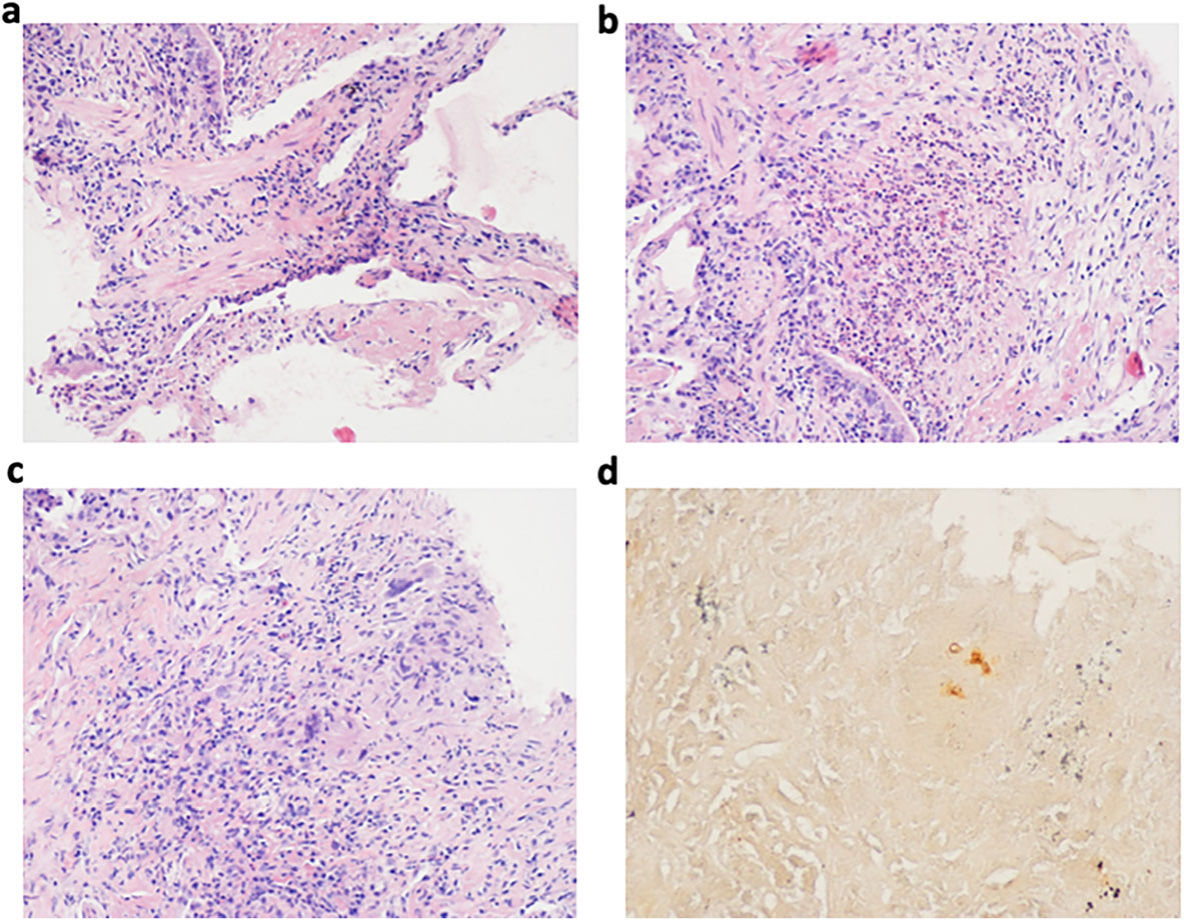
The lung biopsy findings are consistent with the histological pattern of AFOP combined with a fungal infection. **a** Alveolar spaces filled with fibrin and organizing loose connective tissues (hematoxylin and eosin [H&E] stain, × 200). **b** Infiltrations of abundant neutrophils and some lymphocytes and formation of small abscesses (H&E stain, × 200). **c** Epithelioid cell granuloma (H&E stain × 200). **d** Periodic acid-silver methenamine (PAM) staining of a lung biopsy specimen revealing spores (× 400)

**Fig. 4:**
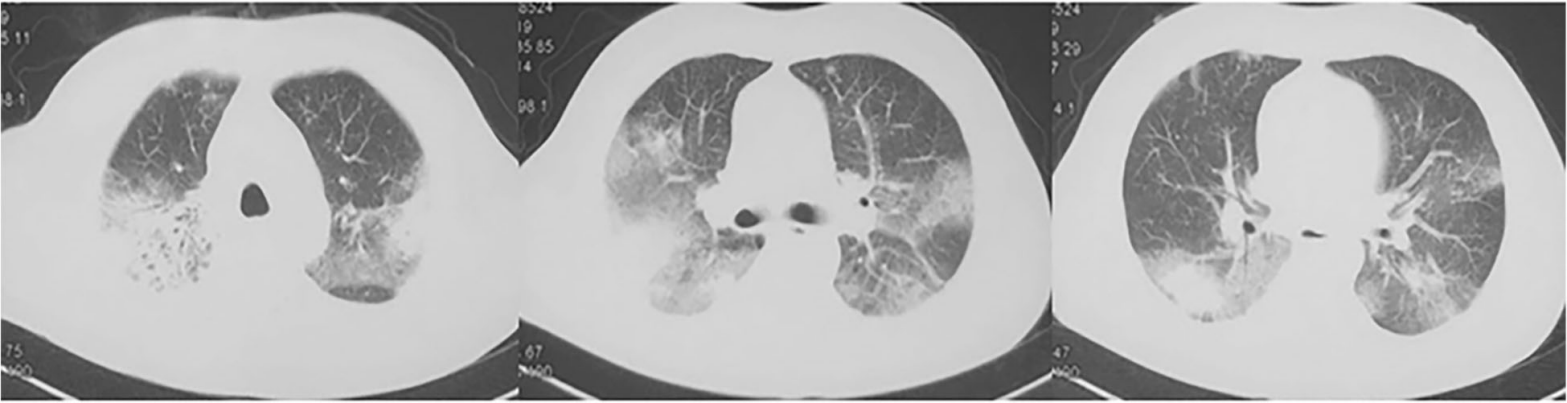
Increasing bilateral diffuse pulmonary infiltrates in both the lungs

Despite these treatments and supportive care, his respiratory status continued to deteriorate, with persistent hyperthermia. Arterial blood gases analysis showed hypoxemia (partial pressure of oxygen (PaO_2_)/fraction of inspired oxygen (FiO_2_) 235 mmHg). The blood culture, staining for acid-fast bacillus in sputum and BAL fluid, and GM test results in BAL fluid were negative. Smear and culture of *Mycobacterium tuberculosis* in sputum and BAL fluid were also negative. Emergency contrast-enhanced chest CT on day 10 revealed bilateral diffuse patchy opacities, multi-focal dense consolidations and bronchial shadows in some lesions **(**Fig. 2**)**. As the antimicrobial drugs were ineffective and organizing pneumonia was considered, the patient was administered with methylprednisolone 40 mg daily; fever subsided, but dyspnea, cough, and hemoptysis underwent progressive worsening. To confirm the diagnosis, we performed a CT-guided percutaneous lung biopsy on day 10. Histologically, the predominant findings were as follows: alveolar spaces filled with fibrin and organizing loose connective tissues involving 70% of the observed region, pulmonary interstitial fibrosis, and small abscesses and epithelioid cell granuloma in the focal area **(**Fig. 3a, b, and c**)**. Result of periodic acid-silver methenamine (PAM) stain was positive **(**Fig. 3 d). The pathological diagnosis was AFOP combined with fungal infection. Methylprednisolone at an increased dose of 40 mg twice daily and voriconazole were continued. However, his condition steadily deteriorated, with continued hypoxemia (the lowest PaO_2_/FiO_2_: 173 mmHg), chest discomfort and exercise intolerance. CT on day 15 showed worsening of the bilateral patchy opacities **(**Fig. 4**)**.

On day 16, a 3-day course of intravenous methylprednisolone was started at 500 mg/d and subsequently, methylprednisolone was continued at 40 mg twice daily. Simultaneously, an antifungal therapy with amphotericin B (dose gradually increased from 10 mg to 40 mg daily) and voriconazole was administered. On day 25, the dose of methylprednisolone was gradually reduced to 30 mg twice daily. Chest CT revealed reduction in the size of the opacity. On day 30, the dose of methylprednisolone was reduced to 40 mg daily. Because of progressive anemia and thrombocytopenia, amphotericin B was discontinued on day 36. On day 37, the patient was switched to oral prednisone (40 mg daily) and voriconazole, with a reduced prednisone dose of 5 mg weekly after discharge from the hospital. Steroid tapering was well-tolerated, with no obvious adverse reactions. Four months after the discharge, chest CT showed complete resolution of the lesions.

**Fig. 5:**
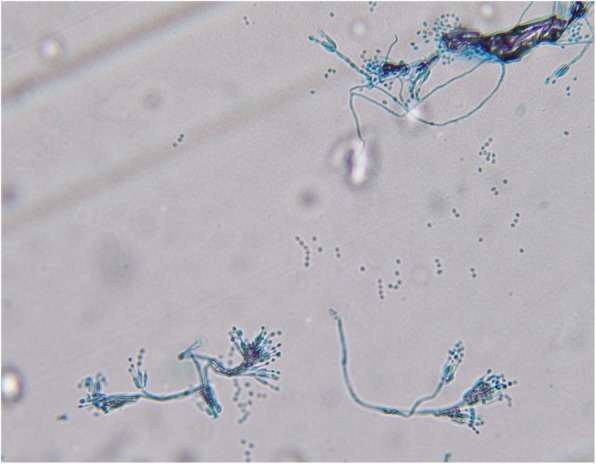
Septate branching hyphae of *P.citrinum* showing ampulliform phialides and spherical conidia (lactophenol blue) (× 100)

The fungal pathogen from the sputum culture was identified as *P. citrinum*. The phialides were ampulliform, bearing well-defined chains of spherical to subspherical conidia, with smooth or finely roughened walls **(**Fig. 5**)**. The fungal pathogen was sent to a reference laboratory (Shanghai Majorbio Bio-pharm Technology Co., Ltd) for identification, and was confirmed as *P. citrinum* with a polymerase chain reaction sequencing analysis. Antifungal sensitivity testing using the Sensititre™ YeastOne™ YO10 Susceptibility Plate (Trek Diagnostics Systems, West Sussex, UK) revealed that the *P.citrinum* isolate demonstrated marked in vitro sensitive to amphotericin B with minimum inhibitory concentration (MIC) of 2 μg/ml and resistance to voriconazole with MIC of > 8 μg/ml.

## Discussion and conclusions

Since the concept of AFOP was proposed by Beasley et al. in a case series involving 17 patients in 2002 [3], AFOP cases have been increasingly reported and recognized. In 2013, AFOP was added to the ATS/ERS classification of IIPs [1]. Patients with AFOP can present with various respiratory signs and symptoms. The common clinical symptoms are fatigue, prolonged fever, cough, dyspnea, and hemoptysis with rapid deterioration leading to respiratory failure. On imaging, various radiographic findings have been described [6]. Patients with AFOP who experience a rapidly progressive course often exhibit diffuse predominant consolidation, ground glass opacities, and multifocal parenchymal abnormalities on imaging.

The diagnosis of AFOP relies on the histologic features of a lung biopsy specimen. Our patient was preliminarily diagnosed with CAP; however, antibacterial, antifungal, and antiviral treatments were ineffective. To confirm the diagnosis, a percutaneous lung biopsy was performed; the pathology was consistent with AFOP. Therefore, clinicians may consider the possibility of AFOP in cases of pneumonia deteriorated rapidly to respiratory failure and refractory to antimicrobial therapy. AFOP is characterized histopathologically by intra-alveolar deposition of fibrin, features of organizing pneumonia with patchy distribution, and absence of the hyaline membrances [3]. The presence of organized fibrin is the predominant histological finding [3, 7].

AFOP is a rare variant of pneumonia with an uncertain etiology. Proposed etiologies include connective tissue disorders, drugs, occupational exposure, immune system disorders, and infections [2, 3, 8–10]. The concomitant occurrence of fungal infection has not been described in patients with AFOP. Our patient appeared to have a fungal infection, which may be *P. citrinum*. *P. citrinum* is ubiquitous in the environment and usually considered as a laboratory contaminant or a non-pathogenic species, as it rarely causes human infection [11–14]. In this case, the patient was initially misdiagnosed as IPA. It is likely that *P. citrinum* shows similar hyaline septate hyphae to *Aspergillus* on direct microscopic examination. Anyway, fluconazole is not an appropriate treatment for Aspergillus at the local hospital.

We considered *P. citrinum* infection in our patient for the following reasons: (i) The growth of *P. citrinum* in five separate sputum cultures from a symptomatic patient with uncontrolled DM prompted careful consideration of a *P. citrinum* pneumonia; (ii) Pathological biopsy revealed small abscesses and granulomatous nodules, and special stain (PAM) result was positive, suggesting fungal pneumonia; (iii) There was no evidence to confirm infection of other organisms. GM test in BAL fluid was negative which is in contrast with previous reports [15, 16]. It’s likely that our patient was non-agranulocytosis and received voriconazole, to which false-negative GM test results have been attributed. Due to the unpleasant experience, the patient refused to undergo tracheoscopy once more. The case presented herein highlight the potential pathogenic role of *P. citrinum* in immunocompromised hosts. Notably, clinicians should be aware of these usual “contaminative” fungi of low pathogenicity and maintain a high index of suspicion in diagnosing this potentially fatal but treatable disease.

Currently, corticosteroid therapy is the most common and effective treatment for AFOP [3, 17, 18], as in this case. There is no consensus on the dosage or duration of corticosteroids. Various regimens have been used depending on the severity and progression of the disease. The long-term effects of corticosteroids have not been established; therefore, monitoring the disease progression is recommended, and reassessments may be necessary. Although there are no standard treatment guidelines for patients with penicilliosis, the first-choice treatment regimen is 0.6 mg/kg/day amphotericin B for 2 weeks, followed by oral itraconazole for 10 weeks [19–21]. Voriconazole should not be considered first-line for the empiric treatment of *P. citrinum* [16]. The high MIC of *P. citrinum* isolate in our patient, which is consistent with previous reports [15, 16, 22], may partly explain why the initial administration of voriconazole was ineffective. Therefore, early antifungal susceptibility testing is essential for appropriate treatment to improve clinical outcomes. Significant relief of symptoms and improvement in radiological findings after the administration of corticosteroids and amphotericin B indicated that the patient received appropriate therapy. However, the patient was treated with corticosteroids and antifungal agents together, it is difficult to understand the effectiveness of antifungal administration.

In summary, we reported an unusual case of AFOP and fungal pneumonia. If clinical symptoms and chest imaging features are unresponsive to broad-spectrum antibiotics, a lung biopsy could be considered and timely performed. As this common “contaminant” can behave as a pathogen in an immunocompromised state, both clinicians and microbiologists should consider the presence of a serious and potentially fatal fungal infection on isolation of *P. citrinum*. Based on this case, it could be speculated that AFOP may occur in association with fungal infection including *P. citrinum*. Our speculation improves the understanding of AFOP. Whether or not *P. citrinum* infection is a risk factor of AFOP needs to be clarified with more investigations and further experiments.

## Data Availability

Data sharing is not applicable to this article as no datasets were generated or analysed.

## Abbreviations

AFOP: Acute fibrinous and organizing pneumonitis
DM: Diabetes mellitus
CT: Computed tomography
IIP: Idiopathic interstitial pneumonia
ATS/ERS: American Thoracic Society/European Respiratory Society
WBC: White blood cell
CRP: C-reactive protein
CAP: Community-acquired pneumonia
GM: Galactomannan
IPA: Invasive pulmonary aspergillosis
BAL: Bronchoalveolar lavage
PaO_2_: Partial pressure of oxygen
FiO_2_: Fraction of inspired oxygen
PAM: Periodic acid-silver methenamine
MIC: Minimum inhibitory concentration
